# Supplementary material for: Polyketide synthase gene clusters in the ericoid mycorrhizal fungus Oidiodendron maius: genome-wide prediction, expression profiling, and functional characterization of knockout mutants
Source: Front Plant Sci. 2026 Sep 16;17:1924430. doi: 10.3389/fpls.2026.1924430 (PMC13623903; doi:10.3389/fpls.2026.1924430)
Supplement: Supplementary file 1 [file SupplementaryFile1.zip › Supplementary Materials/Supplementary Folder 1.zip!/Oidma1_AssemblyScaffolds_Repeatmasked/knownclusterblast/cluster55/jgi.p_Oidma1_21633_mibig_hits.html]

| MIBiG Protein | Description | MIBiG Cluster | MiBiG Product | % ID | % Coverage | BLAST Score | E-value |
| --- | --- | --- | --- | --- | --- | --- | --- |
| AAC49195.1 | pathway\_specific\_transcription\_factor | BGC0000152 | Sterigmatocystin\_biosynthetic\_gene\_cluster | 36 | 105.813953488 | 82 | 3.9e-16 |
| ADM34145.1 | trancriptional\_activator | BGC0001084 | Notoamide\_/\_stephacidin\_biosynthetic\_gene\_cluster | 34 | 100.581395349 | 67 | 1.7e-11 |
| CBF90101.1 | Putative\_Zn(II)2Cys6\_transcription\_factor\_(Eurofung) | BGC0001271 | Emericellin\_biosynthetic\_gene\_cluster | 34 | 100.581395349 | 66 | 3.7e-11 |
| AAS90051.1 | AflR | BGC0000009 | Aflatoxin\_biosynthetic\_gene\_cluster | 33 | 106.976744186 | 66 | 4.9e-11 |
| EAL89341.2 | C6\_transcription\_factor\_(AflR),\_putative | BGC0001403 | Trypacidin\_biosynthetic\_gene\_cluster | 32 | 97.6744186047 | 52 | 5.6e-07 |
